# Experience and Operation of Establishing the Korea Biobank Network for Inflammatory Bowel Disease and Colorectal Cancer

**DOI:** 10.3390/jcm15218485

**Published:** 2026-10-31

**Authors:** Yu-Jin Ko, Mihyang Jeon, Ji-Hyeon Lee, Daeun Kwak, Eun Ju Kim, Hye-Ra Jung, Ji-Hyun Seo, Eun-Young Kim, Eun Soo Kim, Ben Kang, Soo Yeun Park, Man-Hoon Han, An Na Seo

**Affiliations:** 1 Human Biobank, Kyungpook National University Hospital, Daegu 41940, Republic of Korea; xiaoyu1@naver.com (Y.-J.K.); jeonne@naver.com (M.J.); wlgud3669@naver.com (J.-H.L.); dune0560@naver.com (D.K.); ml770@naver.com (E.J.K.); 2 Department of Pathology, Keimyung University School of Medicine, Daegu 42601, Republic of Korea; junghr0519@dsmc.or.kr; 3 Human Resources Bank of Keimyung University Dongsan Hospital, Daegu 42601, Republic of Korea; 4 Department of Pediatrics, Gyeongsang National University, Jinju 52727, Republic of Korea; seozee@hanmail.net; 5 Korea Biobank Network, Gyeongsang National University Hospital, Jinju 52727, Republic of Korea; 6 Department of Clinical Pharmacology, Inje University Busan Paik Hospital, Busan 47392, Republic of Korea; eykimmdphd@gmail.com; 7 Inje Biobank, Inje University Busan Paik Hospital, Busan 47392, Republic of Korea; 8 Department of Internal Medicine, School of Medicine, Kyungpook National University, Daegu 41944, Republic of Korea; dandy813@hanmail.net; 9 Department of Pediatrics, School of Medicine, Kyungpook National University, Daegu 41944, Republic of Korea; benkang@knu.ac.kr; 10 Department of Surgery, Colorectal Cancer Center, School of Medicine, Kyungpook National University, Daegu 41404, Republic of Korea; psy-flower@hanmail.net; 11 Department of Pathology, School of Medicine, Kyungpook National University, Daegu 41405, Republic of Korea; one-many@hanmail.net

**Keywords:** biobank, real-world data, inflammatory bowel disease, colorectal cancer, bioresources, Korea Biobank Network

## Abstract

**Background**: Inflammatory bowel disease (IBD), including Crohn’s disease (CD) and ulcerative colitis (UC), is a chronic condition driven by complex interactions among genetic, microbial, immunological, and environmental factors that substantially affect patients’ lives and increase the risk of colorectal cancer (CRC). Biobanks support IBD research by providing access to human biospecimens and associated data. This study aimed to describe the workflow and infrastructure of the Kyungpook National University Hospital (KNUH) Biobank, summarize the clinical characteristics of participants and the collection and distribution of biospecimens, and outline future development priorities. **Methods**: This descriptive report summarizes the collection and distribution activities of the KNUH Biobank from 1 July 2021 to 31 December 2025, including biospecimens collected at Kyungpook National University Chilgok Hospital and incorporated into its collection. Biospecimens and associated clinical and epidemiological data were collected from consenting participants, processed, and stored according to standard operating procedures. Bioresource distribution required formal application and committee approval. **Results**: The disease-focused collection comprised 1307 participants: 471 with CD (36.0%), 376 with UC (28.8%), 35 with other non-infective gastroenteritis and colitis (2.7%), and 425 with CRC (32.5%). Of these, the mean age at diagnosis was 37.7 years (SD, 23.1), and 849 participants (65.0%) were male. A total of 9577 biospecimen vials were collected from these 1307 participants, excluding the bottom-up collection. Additionally, excluding the bottom-up collection, 2400 vials were distributed through 34 approved applications during the reporting period. **Conclusions**: This report describes the operational framework and collection and distribution activities of the KNUH Biobank. Future priorities include improving clinical data completeness, establishing systematic longitudinal follow-up, and evaluating resource utilization and operational sustainability.

## 1. Introduction

Inflammatory bowel disease (IBD) is a chronic disorder characterized by complex interactions between the gut microbiota and host immune responses; nevertheless, its etiology remains elusive [1,2,3]. The clinical manifestations of IBD vary according to the severity and anatomical location of inflammation [4]. Its symptoms range from mild to severe, with the clinical course being typically characterized by alternating periods of active disease and remission [5]. These clinical characteristics profoundly impair the quality of life of patients with IBD [6].

IBD can be classified into two primary subtypes: Crohn’s disease (CD) and ulcerative colitis (UC). Extensive CD or UC involving the majority of the colorectum is associated with a significantly increased risk of colorectal cancer (CRC) [7]. IBD-associated carcinogenesis is hypothesized to be driven by chronic sustained inflammation within the colorectum, intertwined with a combination of genetic, immunological, infectious, and environmental factors [8]. Both CD and UC are characterized by immune system dysregulation, and the exact mechanisms underlying their pathogenesis have not been fully elucidated. Their onset is widely thought to arise from a complex multifactorial interplay among genetic susceptibility, host microbiota, immunological pathways, and environmental factors that disrupt mucosal homeostasis [4,9].

Genetic susceptibility contributes to the development of both CD and UC [10], and a family history of IBD is an established risk factor [11]. Both CD and UC are chronic diseases associated with substantial morbidity, and patients with colonic involvement have an increased risk of CRC [7,12]. NOD2 (CARD15) [13], IBD5 [14], IL-23 receptor gene [15], and ATG16L1 [16] play a role in CD development [17], whereas Th17 signaling plays a pivotal role in IBD pathogenesis, a link further supported by polymorphisms in the IL-23 receptor and other constituent molecules of the Th17 pathway [15]. The high genetic association in IBD development provides an important direction for developing personalized approaches to IBD diagnosis and treatment.

Advancing research to elucidate IBD pathogenesis and identify novel therapeutic targets has the potential to improve clinical outcomes significantly. Accurately predicting individual disease trajectories and formulating personalized therapeutic strategies depend on the establishment of relationships between biomarkers and clinical phenotypes, which can be achieved by integrating prospective patient data (e.g., disease activity, complications, treatments) with biorepository and multi-omics datasets [18,19]. Therefore, to advance and facilitate large-scale well-structured IBD research, we established an IBD- and CRC-specific biobank at the Kyungpook National University Hospital (KNUH) in collaboration with three other hospitals, which intimately entwined the collection of clinical and epidemiological data, pathological and radiological images, and biological specimens. This biobank was established as an integrated resource to investigate the biological link between intestinal inflammation and CRC. In particular, the mechanistic connection between chronic mucosal inflammation in IBD and colitis-associated cancer provides a clear scientific rationale for encompassing both disease entities within a single biobank [12,20,21]. This integrated framework facilitates the systematic acquisition of diverse biospecimens across the spectrum of inflammatory and neoplastic diseases, thereby fostering translational research into inflammation-driven carcinogenesis and comparative biomarker discovery.

The present study aimed (i) to outline the workflow and information technology infrastructure of the KNUH Biobank within the Korea Biobank Network (KBN), (ii) to describe for the first time the clinical characteristics of the cohorts collected to date, and (iii) to present plans for future expansion and application to advance IBD research by investigators facing challenges in acquiring human biospecimens.

## 2. Materials and Methods

### 2.1. Korea Biobank Project

The National Biobank of Korea (https://biobank.nih.go.kr, accessed on 5 December 2025) has promoted the Korea Biobank Project since 2008 [22], which collects and manages human bioresources that would be highly useful for future healthcare research and development (R&D), including genome-based precision medicine, and distributes bioresources to researchers in the Republic of Korea, thereby facilitating healthcare research and relevant industry development. The KBN has been expending ongoing efforts to standardize and develop domestic biobanks. Various types of biorepositories, such as population-based, disease-oriented, and tissue biobanks, have been reported [23]. As a national fourth phase project, the KBN operated 10 specialized biobank networks (subnetworks) from 2021 to 2025, which were strategically selected to advance disease-specific biobanking for the future according to the changing healthcare R&D environment and research needs and characterized human resource projects for each disease group by linking high-value clinical and epidemiological data [24]. IBD was selected as one of ten disease areas supported by the specialized biobank network. KBN support provides infrastructure for biospecimen collection and management to support IBD biobanking and research. Integrating human biospecimens accrued through this initiative with detailed clinical data would facilitate large-scale investigations into the etiology, prevention, diagnosis, and treatment of IBD. This initiative sought to standardize the acquisition of human bioresources, as well as clinical and epidemiological data, by establishing a systematic network comprising regional hub banks and affiliated partner banks. The KNUH Biobank oversees the base center of the IBD-specific biobank network unit, as well as three other biobanks (namely, Keimyung University Dongsan Hospital, Gyeongsang National University Hospital, and Inje University Busan Paik Hospital), which each collect and store bioresources and manage the data. This IBD-specific biobank network obtains high-value bioresources related to IBD, colorectal dysplasia, and CRC, including comprehensive big data encompassing clinical, epidemiological, and imaging information (endoscopy, magnetic resonance imaging [MRI], computed tomography [CT], digital pathological slide scans, etc.). The present report focuses on the KNUH Biobank collection, including biospecimens collected at Kyungpook National University Chilgok Hospital (KNUCH) and incorporated into that collection, and does not constitute a network-wide analysis of the participating biobanks.

### 2.2. KNUH Biobank

The KNUH Biobank has continuously participated in the KBN project since its inception in the first phase. This report focuses exclusively on biospecimens and associated data collected during a specific period of the fourth phase, spanning from 1 July 2021 to 31 December 2025. Subsequent participation in the fifth phase is being carried forward by other investigators and remains beyond the scope of this study. Its primary objective is to collect, store, and distribute biospecimens and associated clinical and epidemiological data from patients and healthy controls, while linking these resources for research use. Participants were recruited from clinical departments at two affiliated academic hospitals: Kyungpook National University Hospital (KNUH) and KNUCH in Daegu, Republic of Korea. Written informed consent for the collection and storage of clinical information and biospecimens was obtained from all participants whose biospecimens were deposited in the KNUH Biobank. Participant enrollment and informed consent were conducted prospectively during routine clinical practice. Patients who voluntarily agreed to participate provided written informed consent prior to biospecimen collection. Following acquisition and banking, no participants withdrew consent. Because participant enrollment was embedded within routine outpatient and inpatient clinical workflows and was based on voluntary opt-in participation, a screening log for individuals approached who declined was not formally tracked; consequently, exact recruitment and consent rates could not be determined.

### 2.3. Informed Consent

In accordance with the Enforcement Rules of the Bioethics and Safety Act and related guidelines in the Republic of Korea, the KNUH Biobank obtains informed consent for the following activities from participants after they have received a full explanation: (i) the KNUH Biobank provides secure collection, long-term storage, high-throughput analysis, and strategic distribution of double-pseudonymized deep clinical, epidemiological, and imaging data and biological specimens; (ii) these assets are made available to academic, non-academic, and commercial research entities worldwide; and (iii) this infrastructure enables the seamless linkage of biobank research inputs with routine real-world clinical data. By providing informed consent, participants authorized the use of their double-pseudonymized data and biospecimens for research within the scope of their consent and subject to the applicable review and approval procedures. Participants could withdraw their consent at any time without providing a reason. Under the biobank’s procedures, data and biospecimens would be deleted and discarded if consent were withdrawn. In accordance with Article 36 (“Deliberation on Human Material Research”) under the Bioethics and Safety Act of the Republic of Korea, the research plan for the fourth-phase project was reviewed and approved by the Institutional Review Boards (IRBs) of KNUH (approval no. 2021-05-051) and KNUCH (approval no. 2021-06-029). Biospecimen collection at each hospital commenced only after approval by its respective IRB: 1 July 2021 for KNUH, and 23 September 2021 for KNUCH. The reporting period ended on 31 December 2025. This study was conducted in strict accordance with the ethical principles embodied in the Declaration of Helsinki. Written informed consent was obtained from all participants before sample collection and data acquisition.

### 2.4. Patient Selection and Biospecimen Collection

The fourth phase of the KBN project began on 31 March 2021. The present report includes only data and biospecimens collected from 1 July 2021 to 31 December 2025. Standardized IBD-related clinical and epidemiological data and biospecimens—including serum, fresh-frozen tissue specimens (including colonic biopsy), and stool samples—were obtained. Diagnoses of CD and UC were established by the treating clinicians based on clinical findings and relevant investigations, including histopathological findings. The Korean Standard Classification of Diseases (KCD) codes were used to classify and record these clinical diagnoses rather than as standalone diagnostic criteria. The IBD cohort comprised patients diagnosed with CD (KCD code: K50) or UC (KCD code: K51). Patients with clinically suspected but unconfirmed IBD at initial presentation were also eligible for biospecimen collection. Biospecimen collection was not restricted to a predefined disease stage or treatment status. Disease phenotype classification and treatment status at sampling were not systematically captured in the dataset used for this report. These cases were prospectively registered under other non-infective gastroenteritis and colitis (Other GE/colitis, KCD code: K52), capturing the diagnostic ambiguity inherent to early-stage disease rather than a definitive IBD diagnosis. While Other GE/colitis was retained as an independent diagnostic classification, the resulting biospecimen inventories (including blood samples) were aggregated across the Crohn’s disease, ulcerative colitis, and Other GE/colitis cohorts. For this report, the CRC cohort comprised patients diagnosed with CRC (KCD codes: C18–C20). Although colorectal dysplasia was included in the broader network’s collection scope, patients with dysplasia alone were not included in the present analysis. The biobank comprised an IBD-related collection, including patients registered with CD, UC, or K52, and a separate CRC cohort. Additionally, the bottom-up collection primarily comprised biospecimens and associated clinical data from patients with conditions other than IBD, Other GE/colitis, or CRC encountered during routine clinical care and individuals attending the hospital for health check-ups who had neither IBD nor cancer. The latter were designated as healthy controls for this biobank. All participants provided written informed consent before biospecimen collection.

### 2.5. Patient Privacy and Data Pseudonymization

In accordance with Article 44 (“Obligations of Human Material Banks”) under the Bioethics and Safety Act of the Republic of Korea, guidelines on personal information protection, including schemes for human material anonymization, have been established for the biobank, as prescribed by the Ministry of Health and Welfare. Information related to human bioresources, except for identification information, is managed separately from human bioresources. An information protection system has been devised to protect the providers’ personal identification information against leakage by filtering necessary information when inquiring about clinical and epidemiological data. Clinical and epidemiological data underwent double pseudonymization. Hospital patient identifiers were first replaced with study identifiers, which were subsequently replaced with biobank identifiers (bCODEs) when the data were loaded into the KBN common data model.

### 2.6. Procedures for Bioresource Collection: Processing and Storage

**Figure 1:**
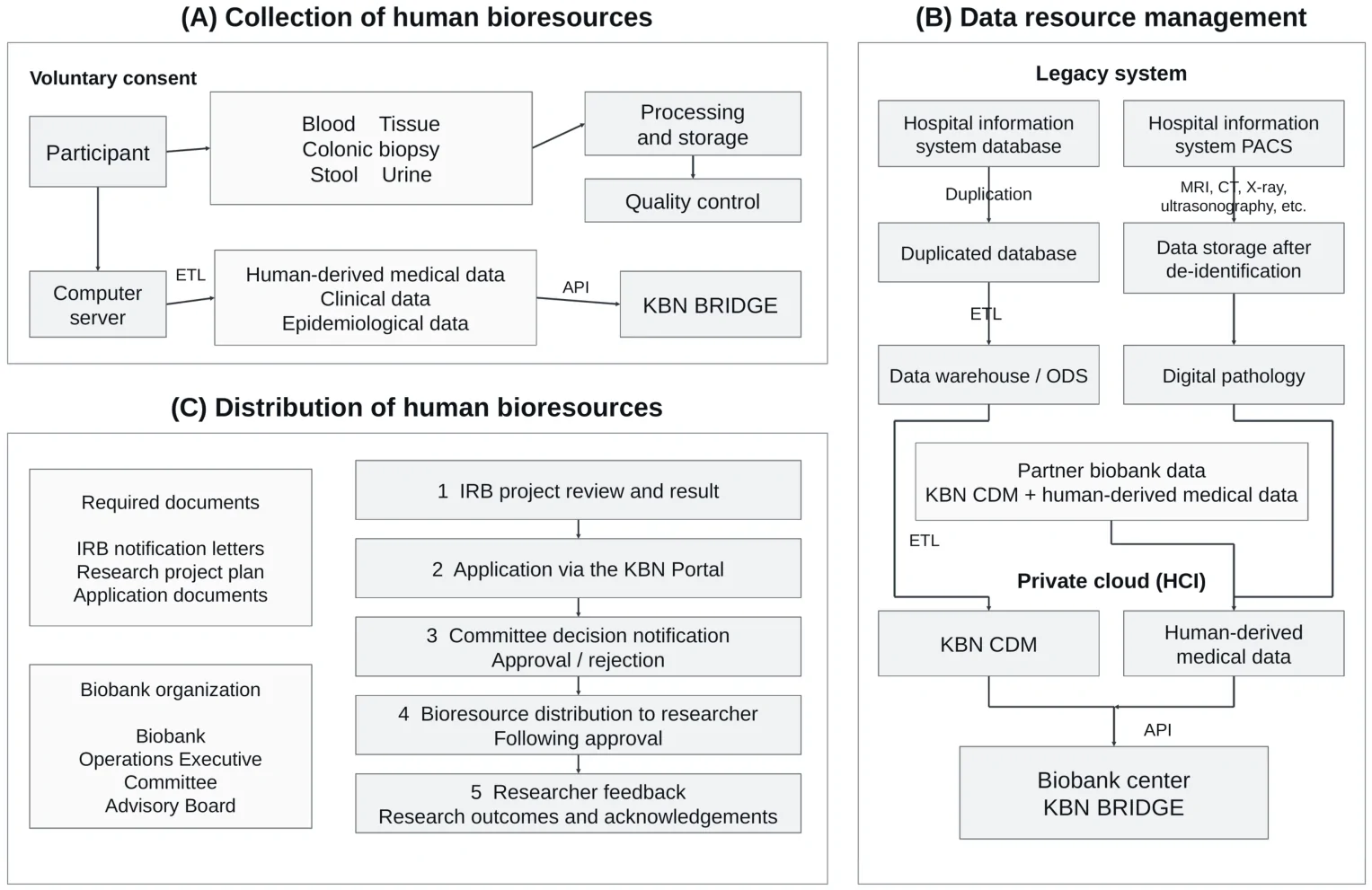
Schematic operating flowchart of an inflammatory bowel disease-specific biobank. (**A**) Collection of human bioresources. (**B**) Data resource management. (**C**) Distribution of human bioresources. The IBD-specific biobank network unit (base bank and partner biobank) collects and manages human bioresources and related data resources and supports the distribution of bioresources. Biological samples such as blood, tissue, and stool are systematically collected from IBD patients, and samples are processed and stored for future research. Clinical and epidemiological data associated with each biological sample, as well as medical imaging data, are also safely managed. The data are stored in a de-identified and searchable database (Korea Biobank Network) to protect patient privacy and facilitate research. Researchers can request and receive samples for their research. The distribution process involves reviewing the research proposal, shipping the samples to researchers, and providing feedback on the results of using human bioresources. ETL, Extract, transform, load; API, application programming interface; KBN, Korea biobank network; BRIDGE, Biobank research information and digital image exchange; PACS, picture archiving and communication system; MRI, magnetic resonance imaging; CT, computed tomography; ODS, operational data store; CDM, common data model; HCI, hyperconverged infrastructure; IRB, institutional review board.

Human bioresource collection (Figure 1A) was processed as follows. Residual biospecimens were collected during medical procedures to acquire biological resources for the KNUH Biobank. The samples were placed in sterile vials with appropriate buffers and were shipped under strictly controlled and monitored conditions (e.g., sample type and storage). Upon arrival at the biobank, the samples were labeled with biobank identifiers (bCODEs) and stored. An aliquoting procedure was implemented to reduce freeze–thaw cycles of samples, create backups, and facilitate easier sharing of samples without thawing [23]. Standard operating procedures (SOP) for biospecimen processing and quality control were developed with reference to International Society for Biological and Environmental Repositories (ISBER) Best Practices, KS J ISO 20387, and the KBN SOPs issued by the National Biobank of Korea [25]. The processing methods are shown according to the type of resources collected in the biobank in Table 1.

For assays requiring subsampling, participants contributing each specimen type were randomly selected, and their biospecimens underwent quality control (QC) testing. The planned inspection ratios indicate the minimum proportion of participants contributing each specimen type whose biospecimens were designated for inspection. Acceptance criteria were applied according to specimen type and the relevant assay: an absorbance ratio at 260 and 280 nm (A260/A280) of 1.6–2.0 for DNA purity, an RNA integrity number (RIN) of ≥4 for RNA integrity, the absence of visible hemolysis in applicable blood-derived specimens, and the absence of detectable target-specific bands following polymerase chain reaction (PCR) testing for bacterial and mycoplasma contamination. Histopathologic examination was performed to assess tissue adequacy using mirror-image hematoxylin and eosin (H&E)-stained sections for fresh-frozen tissue specimens. For pre-analytical quality control, the standardized processing and storage conditions for each specimen type are summarized in Table 1. Available pre-analytical information was recorded using Standard PREanalytical Code (SPREC)-based fields v.3.0 [26]. Warm and cold ischemia times were not captured in the recording system used for this collection. Routine QC records were compiled, and the resulting quality metrics across specimen types are described in the Results Section.

### 2.7. Collection of Clinical, Epidemiological, and Medical Imaging Data

The KNUH Biobank collects standardized multidimensional clinical data encompassing baseline demographic characteristics, medical history, diagnostic information, and prescription records. This database integrates laboratory test results, human-derived medical imaging data (including endoscopy, MRI, and CT), and clinical observations recorded during routine care. The clinical, epidemiological, and medical imaging data of patients were processed as follows (Figure 1B): (i) clinical and epidemiological data were extracted from the hospital’s integrated medical information system to collect human bioresource-related information; (ii) a replica database was created using Oracle Active Data Guard (Oracle Corp., Austin, TX, USA) to centralize the clinical and epidemiological data; (iii) the replicated data were transferred to the data warehouse Operational Data Store (ODS) and subsequently converted to fit the table definition document structure of the KBN common data model provided by the KBN central network; (iv) they were stored in a private cloud (HCI) and transmitted to the KBN Biobank Research Information and Digital Image Exchange (KBN BRIDGE) through an application programming interface.

In addition to the 88 common clinical items defined by the KBN common data model, the IBD-specific biobank developed a data collection framework comprising 111 supplementary clinical and epidemiological items. Together, these items covered participant demographics, diagnoses, family history, physical examination findings, and previous and current treatments. Inclusion in the framework did not imply that every item was available for every participant. Medical imaging data were downloaded from the hospital’s picture archiving and communication system (PACS), de-identified, and stored on a biobank server (HCI). Data records were tracked using a management ledger to reduce identification and linkage errors. Standardized clinical and epidemiological data were managed through the KBN central platform to support access for academic and industrial research, subject to applicable consent and formal application and approval procedures.

### 2.8. Management of Digital Pathological Image Data

Tissue specimens collected for patient diagnosis were processed into routine formalin-fixed paraffin-embedded tissue blocks and were subsequently sectioned and used to generate H&E-stained slides. Representative slides were selected and scanned using Aperio GT 450 (Leica Biosystems, Vista, CA, USA). Scanned digital pathological images were de-identified using Whole-Slide Image DeIdentifier v2 (Humintec, Suwon-si, Republic of Korea) for Aperio GT 450, linked to the corresponding bCODEs, and stored.

### 2.9. Procedures for Bioresource Distribution

The distribution of collected human bioresources was processed according to the procedure shown in Figure 1C. Researchers were required to obtain approval of their research plans from the relevant institutional review board before applying for bioresources. After the researcher had prepared the necessary documents (IRB notification letters, research project plans, and application documents) and applied through the KBN portal, the biobank decided on the distribution through the Operations Executive Committee. For reporting purposes, one distribution case was defined as one approved application for bioresource distribution, regardless of the number of donors or biospecimens included in that application. Bioresources were distributed to researchers in response to approved deliberations and shipped to researchers in accordance with our SOP (e.g., temperature and time during transportation). Research outcomes (papers, patents, etc.) using distributed resources were tracked to monitor bioresource utilization.

### 2.10. Statistical Analysis

Descriptive statistics were used to summarize participant characteristics and quality control results. Continuous variables were presented as mean ± standard deviation (SD) or median with interquartile range (IQR), as appropriate. Categorical variables were presented as counts and percentages. Denominators for percentages are specified in the table footnotes. Data availability was calculated relative to the relevant participant group, whereas category percentages were calculated among participants with available data unless otherwise specified. Analyses were conducted using SPSS software (version 18.0; IBM Corp., Armonk, NY, USA).

## 3. Results

### 3.1. State of Biospecimen Processing, Storage, and Management

The annual collection status of biospecimens during the reporting period is summarized in Table 2. A total of 1307 participants contributed biospecimens during the reporting period. Of the 1307 participants, 847 had IBD (CD: *n* = 471, 55.6%; UC: *n* = 376, 44.4%), 35 had Other GE/colitis, and 425 had CRC. Through the bottom-up approach, biospecimens were collected from 15,608 participants, including patients with conditions other than IBD or CRC and healthy controls. Biospecimens from the 1307 participants were organized into matched sets comprising available specimen types from the same participant, such as blood, buffy coat, serum, stool, and tissue, to facilitate their use in diverse research applications (Table 3). Biospecimens failing to meet the required quality standards were subject to exclusion and disposal in accordance with the biobank’s SOPs.

Quality control results are summarized by specimen type in Table 4, including the actual inspection proportions, test results, and pass counts and rates for the predefined acceptance criteria. Visual inspection was performed on specimens from all participants who contributed plasma, serum, or urine, with pass rates ranging from 83.9% to 96.9%. Failure rates upon visual inspection were 13.6% for plasma and 16.1% for serum, primarily attributable to hemolysis and lipemia. DNA purity acceptance rates were 95.8% to 96.0% for whole blood and buffy coat specimens, with median A260/A280 ratios ranging from 1.86 to 1.87. Tissue RNA exhibited a median RIN of 7.4 (interquartile range [IQR], 7.0–8.0), and 97.5% of tested samples met the acceptance criterion of RIN ≥ 4. No bacterial or mycoplasma contamination was detected in any of the samples tested by PCR.

### 3.2. State of Clinical and Epidemiological Data Management

The demographic, clinical, and epidemiological characteristics of the participants are summarized in Table 5. The disease-focused collection comprised 1307 participants: 847 with IBD (471 with CD and 376 with UC), 35 with Other GE/colitis, and 425 with CRC. Overall, 849 participants (65.0%) were male, and all were of Asian ethnicity. The mean age at diagnosis was 37.7 ± 23.1 years overall and 19.3 ± 11.1 years for CD, 16.8 ± 10.2 years for Other GE/colitis, 37.1 ± 18.0 years for UC, and 63.7 ± 11.0 years for CRC.

Data on smoking status and body mass index (BMI) were available for 60.6% (*n* = 792) and 34.6% (*n* = 452) of participants, respectively, with availability differing by cohort (Table 5). Among those with documented smoking status, 88.4% (*n* = 700) were never-smokers. Alcohol consumption history was recorded for 612 participants (46.8%), of whom 383 (62.6%) reported a history of alcohol consumption. The corresponding proportions were 97.0% (319/329) in CRC, 55.2% (48/87) in UC, and 8.2% (16/195) in CD. Among participants with CD, disease activity scores were available for 411 (87.3%): pediatric Crohn’s disease activity index (PCDAI) scores for 360 participants younger than 18 years and Crohn’s disease activity index (CDAI) scores for 51 participants aged 18 years or older. These represented 76.4% and 10.8% of all 471 participants with CD, respectively. Nancy histological index scores for participants with UC [27] were recorded alongside biospecimen collection but are not reported here because the data were incomplete.

### 3.3. Collection and Provision of Medical Imaging and Digital Pathological Data

For the reporting period, the KNUH Biobank was utilized to manage various medical imaging data and biospecimens from participants. Medical imaging modalities included endoscopy, X-ray, CT, MRI, and ultrasonography, when available. Medical imaging and digital pathology data, when available, were collected and linked to biospecimens and associated clinical and epidemiological data from the same participants. No participants contributed imaging data alone. In the case of digital pathology, H&E-stained slides produced for diagnosis were scanned separately and stored as .svs files, enabling researchers who need them to use them as resources. All collected imaging data were de-identified by removing personally identifiable information before storage and distribution. Efforts were made to enhance the research value of human bioresources through the integrated collection and management of these diverse data. Among the 1307 enrolled participants, multi-modal diagnostic data were widely available: endoscopy was documented in 589 participants, digital pathology in 463, MRI in 308, and CT in 280, whereas X-ray and ultrasonography data were available for 43 and 22 participants, respectively. Individual participants could contribute data to more than one modality (Table 6).

### 3.4. State of Bioresource Distribution

During the reporting period, the KNUH Biobank distributed 12,774 vials through 109 approved distribution applications. Annual distribution volumes fluctuated, ranging from 1040 to 4111 vials (Table 7). Various bioresources (blood, fresh frozen tissue, and stool), medical and pathological images, and clinical and epidemiological data were distributed based on researcher demand for specific research projects. Specifically, for IBD- and CRC-related bioresources, the number of distributions was four in 2021, six in 2022, ten in 2023, seven in 2024, and seven at the end of 2025. During the same period, the total distribution of bottom-up collection bioresources was 75. The number of approved distribution applications decreased from 10 in 2023 to 7 in 2024. Only IBD-related biospecimens were distributed in 2021 and 2022, whereas both IBD- and CRC-related biospecimens were distributed during 2023–2025.

Biomarker development was the most frequently represented research topic among approved distribution applications (*n* = 37). Other topics included diagnostic technology development, functional studies of disease-related genes and proteins, disease model development, and targeted therapeutic development (Table 8). Research outputs from studies using bioresources distributed by the KNUH Biobank, including those from the bottom-up collection, have been reported [28,29,30,31,32,33,34,35,36,37]. The biobank routinely tracks these scholarly outputs to monitor bioresource utilization.

## 4. Discussion

The KNUH Biobank was established to collect and link biospecimens with clinical, epidemiological, and imaging data to support research on IBD and CRC. The KNUH Biobank aims to support translational gastrointestinal research by collecting biospecimens and associated data from patients with IBD, CRC, and other conditions, as well as healthy controls. This study describes its operational framework and collection and distribution activities within the KBN. Ongoing efforts focus on improving resource availability, data completeness, and quality management to support future studies of disease mechanisms and potential diagnostic and therapeutic approaches.

Although historically characterized as a disease prevalent in industrialized Western nations, the incidence and prevalence of IBD have increased rapidly in the Republic of Korea over the past few decades. In 2020, the Korean Association for the Study of Intestinal Diseases (KASID) published an IBD official fact sheet using the National Health Insurance Service database to delineate these epidemiological trends [38,39]. According to the KASID 2020 fact sheet, the total number of patients with IBD expanded substantially from 2010 to 2019; specifically, CD cases increased from 7700 to 18,463, whereas UC cases increased from 16,136 to 37,439 [39]. These data represent 2.4-fold and 2.3-fold increases in the respective cohorts over the 10-year period [39]. Regionally, the prevalence of CD in Daegu was the highest in the country at 48.4 per 100,000 individuals, and the prevalence of UC also reached a remarkably high level at 70.7 per 100,000 individuals [39]. These regional disease burdens provided a rationale for establishing an IBD-focused biobank with a complementary CRC collection to support comparative research on inflammation-associated carcinogenesis. During the fourth phase of the KBN project, the KNUH Biobank participated in a disease-specific network supporting the standardized collection of biospecimens and associated clinical and epidemiological data. The present report describes the collection and distribution activities of the KNUH Biobank from 1 July 2021 to 31 December 2025, including biospecimens collected at KNUCH and incorporated into the KNUH Biobank collection. The disease-focused collection comprised 1307 participants: 471 with CD, 376 with UC, 35 registered with Other GE/colitis, and 425 with CRC. Thus, 847 participants had CD or UC, while participants registered with non-infective gastroenteritis and colitis were reported separately. CRC was included as a distinct disease cohort rather than a control group. Associated clinical and epidemiological information was collected, although data completeness varied across individual variables. Where available, medical imaging and digital pathology data were linked to biospecimens from the same participants. While these integrated resources provide a platform to investigate correlations between clinical phenotypes and tissue-level findings, associations with disease progression and therapeutic response were beyond the scope of the present report. As part of the bottom-up collection, specimens were additionally gathered from participants with other conditions as well as individuals undergoing routine health check-ups who had neither IBD nor cancer, with the latter designated as healthy controls.

Biobanks play a pivotal role in promoting practical research utilization by collecting resources and facilitating their accessibility to researchers. In the KNUH biobank, the number of approved applications for IBD- and CRC-related bioresources increased from four in 2021 to ten in 2023, followed by seven in both 2024 and 2025. The KBN encourages biobanks to promote their collections to researchers to increase awareness of available resources and encourage applications for bioresource access. Interestingly, approved distribution applications at KNUH biobank also included research involving machine learning and deep learning (Table 8). Linking biospecimens with medical imaging, digital pathology, and clinical data may support such research. However, the suitability of these resources for specific artificial intelligence (AI) applications and their effects on model performance were not evaluated in this study. This report provides an overview of the establishment, operational framework, and biospecimen collection activities of the KNUH Biobank.

Recent advancements in AI, big data, and information technology have revolutionized the use of biospecimens [40]. Biobanks may support machine learning and deep learning research by providing access to biospecimens and associated clinical and imaging data. Notably, even in the absence of physical biological samples, pairing high-quality medical and digital imaging data with clinical and epidemiological data, as well as genetic and molecular data, may offer substantial research value for the advancement of AI technology, specifically for the development and refinement of image analysis algorithms [41,42,43]. For instance, a previous study developed a deep learning model using stool photographs to predict endoscopic mucosal inflammation in patients with UC [44]. The accumulation and analysis of large-scale datasets may support the development and validation of AI models across healthcare and life sciences [45]. When physical biospecimens are difficult to obtain, existing digital data can provide resources for AI-driven research without requiring additional specimen collection [43,46]. Accordingly, the fifth phase of the KBN project, which began on March 1, 2026, places greater emphasis on collecting medical imaging, digital pathology, and genetic and molecular data while continuing biospecimen collection.

KBN promotes the standardization and infrastructural development of biobanks across the Republic of Korea through competitive proposal evaluations and partial funding for operational and research expenditures. Illustrating the tangible outcomes of this initiative, the Korea Oral Biobank Network established and published standardized operating procedures for collecting, processing, and preserving oral biospecimens [47]. A KBN working group developed consensus definitions for subcategories of normal controls to support the selection of appropriate blood samples for research [48]. Concurrently, several studies highlight the development of training methodologies in compliance with ISO 20387, ensuring rigorous quality control and institutional standardization [49]. While these ongoing efforts continue, several challenges remain to optimize the role of biobanks. One significant issue is the standardization of data and sample collection across different institutions. As biobanks expand, ensuring the consistency and quality of samples and data is crucial for maintaining the integrity of research findings [50]. Furthermore, it is paramount to address ethical considerations pertaining to patient consent, data privacy, and the utilization of samples in research. Balancing research access with the protection of participants’ rights, privacy, and consent is an important consideration in the ethical and sustainable operation of biobanks [51,52].

Several limitations of this study warrant consideration. First, participant enrollment during routine clinical care relied on voluntary informed consent, which may have introduced selection and participation biases. Second, our findings represent a unified biobank collection across two affiliated academic hospitals within a single geographic region, potentially limiting generalizability to broader demographic settings. Third, although the collection supports IBD research, the available sample sizes may limit analyses of specific disease subgroups and phenotypes. Fourth, clinical data completeness varied across variables: smoking status, alcohol consumption history, and body mass index were available for 60.6%, 46.8%, and 34.6% of participants, respectively. This missingness restricted detailed cohort characterization and may introduce bias if participants with available data differ from those with missing data. The availability of PCDAI and CDAI scores was reported relative to all participants with CD rather than the respective age-eligible populations, limiting interpretation of age-specific data completeness. Additionally, BMI was presented using the same fixed categories across all ages; these categories should not be interpreted as pediatric weight-status classifications. Furthermore, the lack of systematic longitudinal follow-up precludes evaluating dynamic changes in disease activity, therapeutic regimens, or long-term clinical outcomes. In addition, healthy controls were included in the bottom-up collection, but their number and clinical characteristics were not reported separately. Finally, operating costs, cost-effectiveness, and long-term financial sustainability were not assessed.

## 5. Conclusions

In conclusion, this report describes the operational framework, biospecimen acquisition, and distribution profile of the KNUH Biobank during the specified reporting period. By integrating high-quality biospecimens and multi-modal clinical data from participants with IBD, CRC, and non-IBD conditions alongside healthy controls, this bioresource provides a valuable platform for translational research. Future efforts should prioritize establishing systematic longitudinal follow-up cohorts, enhancing clinical data completeness, and maintaining rigorous standardization and quality assurance. Furthermore, comprehensive evaluations of bioresource utilization patterns and operational sustainability will be essential to maximize the long-term scientific impact of the biobank.

## Figures and Tables

**Table 1:** Sample processing and storage conditions for bioresources in the biobank.

Type of Sample	Primary Container	Short-Term Storage/ Transport Condition	Centrifuge	Long-Term Storage	SPREC Code ^•^
**Blood** **(Whole)**	EDTA tube	2–10 °C/on ice within 2 h	─	−80 °C	BLD-PED-B-N-N-A-D
**Buffy coat**	EDTA tube	2–10 °C/on ice within 2 h	2–10 °C, 10–15 min, 2500 rpm	−80 °C	BUF-PED-B-C-N-A-D
**Plasma**	EDTA tube	2–10 °C/on ice within 2 h	2–10 °C, 10–15 min, 2500 rpm	−80 °C	PL1-PED-B-C-N-A-D
**Serum**	SST	2–10 °C/on ice within 2 h	2–10 °C, 10–15 min, 2500 rpm	−80 °C	SER-SST-B-C-N-A-D
**Stool**	Cryotube	─	─	−80 °C	STL-CRT-X-N-N-N-D ^†^
**Tissue**	Biopsy/ Surgical excision	LN2 in vacuum container	─	−196 °C LN_2_	TIS-BPS-N-N-SNP-N-C TIS-SRG-N-N-SNP-N-C
**Urine**	PE tube	2–10 °C/on ice within 2 h	─	−80 °C	URN-PET-B-N-N-N-D

SPREC, Standard PREanalytical Code; EDTA: ethylenediaminetetraacetic acid; SST: serum separator tube; PE: polyethylene; LN2: liquid nitrogen. ^•^ SPREC designations were assigned to each vial. ^†^ Stool specimens were transported under refrigerated conditions.

**Table 2:** Number of participants by collection category in the biobank, 2021–2025.

Type	The Number of Participants ^†^					
	2021	2022	2023	2024	2025	Total
**Inflammatory bowel disease**	131 (945) *	319 (2713)	221 (2585)	101 (621)	75 (468)	847 (7332)
**Crohn’s disease** **(CD)**	66 (625)	204 (2010)	138 (1943)	49 (318)	14 (89)	471 (4985)
**Ulcerative colitis** **(UC)**	65 (320)	115 (703)	83 (642)	52 (303)	61 (379)	376 (2347)
**Other noninfective** **g** **astroenteritis and colitis** **(Other GE/colitis)**	6 (105)	27 (115)	─	─	2 (2)	35 (222)
**Colorectal cancer** **(CRC)**	6 (37)	76 (392)	109 (517)	114 (762)	120 (315)	425 (2023)
**Bottom-up collection**	1804 (10,939)	3309 (21,735)	3272 (21,917)	3818 (17,194)	3405 (14,911)	15,608 (86,696)

^†^ The reporting period was 1 July 2021 to 31 December 2025. * The number in parentheses is the total number of vials.

**Table 3:** Matched biospecimen sets from 1307 participants in the biobank, 2021–2025.

Type	The Number of Participants ^†^					
	2021	2022	2023	2024	2025	Total
**Blood (Whole)**	65 (109) *	124 (219)	129 (168)	68 (68)	24 (35)	410 (599)
**Buffy Coat**	5 (6)	107 (114)	172 (203)	106 (147)	60 (68)	450 (538)
**Plasma**	39 (49)	145 (166)	159 (216)	106 (427)	59 (115)	508 (973)
**Serum**	96 (143)	290 (454)	158 (415)	64 (119)	52 (90)	660 (1221)
**Stool**	107 (278)	217 (854)	166 (639)	66 (201)	42 (97)	598 (2069)
**Tissue** **(Normal)**	92 (473)	213 (1178)	219 (810)	96 (218)	51 (87)	671 (2766)
**Tissue** **(Abnormal)**	27 (29)	34 (43)	116 (369)	38 (52)	40 (56)	255 (549)
**Tissue** **(Tumor)**	─	73 (192)	101 (257)	58 (140)	29 (66)	261 (655)
**Urine**	─	─	3 (25)	11 (11)	117 (171)	131 (207)
**Total**	143 (1087)	422 (3220)	330 (3102)	215 (1383)	197 (785)	1307 (9577)

^†^ Participants may be counted in more than one specimen-type category. The total represents 1307 participants across the CD, UC, Other GE/colitis, and CRC categories. Numbers in parentheses indicate vial counts, totaling 9577, consistent with Table 2. * The number in parentheses is the total number of vials.

**Table 4:** Quality control inspection methods, inspection ratios, and results for bioresources in the biobank.

Type of Sample	Evaluation Contents	Planned Ratio ^†^	Inspected, *n*/*N* (%)	Result, Median (IQR) ^‡^	Passed, *n* (% of Inspected) ^§^
**Blood (** **w** **hole)**	DNA purity assessment	≥3%	25/410 (6.1%)	1.86 (1.84–1.90)	24 (96.0%)
	PCR-based bacterial detection (bacteria, mycoplasma)	≥10%	55/410 (13.4%)	—	55 (100%)
**Buffy coat**	DNA purity assessment	≥3%	48/450 (10.7%)	1.87 (1.85–1.91)	46 (95.8%)
	PCR-based bacterial detection (bacteria, mycoplasma)	≥10%	46/450 (10.2%)	—	46 (100%)
**Plasma**	Visual inspection	100%	508/508 (100%)	—	439 (86.4%)
**Serum**	Visual inspection	100%	660/660 (100%)	—	554 (83.9%)
**Tissue (all) ^¶^**	RNA integrity	≥3%	40/1187 (3.4%)	7.4 (7.0–8.0)	39 (97.5%)
	Histopathologic examination	≥95%	1130/1187 (95.2%)	—	1109 (98.1%)
**Urine**	Visual inspection	100%	131/131 (100%)	—	127 (96.9%)

*n*/*N*, number of participants whose specimens underwent the specified QC assessment/total number of participants contributing that specimen type. For tissue QC, counts were based on participant–tissue-type combinations, as defined below. One vial per biospecimen was randomly selected for DNA, bacterial, and RNA testing; visual inspection was performed on specimens from all participants contributing the relevant specimen types; histopathologic examination was performed per tissue biospecimen. A260/A280, ratio of absorbance at 260 and 280 nm; IQR, interquartile range; PCR, polymerase chain reaction; RIN, RNA integrity number. ^†^ Minimum proportion of participants contributing each specimen type whose biospecimens were designated for QC inspection. ^‡^ A260/A280 for DNA purity; RIN for RNA integrity; —, qualitative pass/fail assessment. ^§^ Number (%) meeting the acceptance criteria (A260/A280 1.6–2.0; RIN ≥ 4; no detectable target-specific bands following PCR; no hemolysis, lipemia, turbidity, or hematuria on visual inspection; tissue compatibility), according to the Korea Biobank Network Standard Operating Procedures. Counts use the same units as the corresponding inspection counts, and percentages are calculated using the number inspected as the denominator. ^¶^ For tissue QC, each participant–tissue-type combination was counted as one unit. The denominator comprised 671 normal, 255 abnormal, and 261 tumor tissue units (total, 1187; Table 3). Participants contributing more than one tissue type were counted separately for each type. Histopathologic examination was not performed for 57 tissue units because of insufficient stored tissue (e.g., small biopsy specimens). Quality control inspections were conducted following the Korea Biobank Network Standard Operating Procedures published by the National Biobank of Korea.

**Table 5:** Demographic characteristics from clinical and epidemiological data of inflammatory bowel disease, other noninfective gastroenteritis and colitis, and colorectal cancer participants collected with bioresources in the biobank from 2021 to 2025.

Characteristic	Total	CD	UC	Other GE/ Colitis	CRC
Participants, *n*	1307	471	376	35	425
Sex, *n* (%)					
Male	849 (65.0)	333 (70.7)	228 (60.6)	22 (62.9)	266 (62.6)
Female	458 (35.0)	138 (29.3)	148 (39.4)	13 (37.1)	159 (37.4)
Age at diagnosis, mean ± SD (years) ^†^	37.7 ± 23.1	19.3 ± 11.1	37.1 ± 18.0	16.8 ± 10.2	63.7 ± 11.0
Ethnicity: Asian, *n* (%)	1307 (100.0)	471 (100.0)	376 (100.0)	35 (100.0)	425 (100.0)
Smoking status, available *n* (%) ^§^	792 (60.6)	291 (61.8)	148 (39.4)	17 (48.6)	336 (79.1)
Current smoker, *n* (%)	40 (5.1)	5 (1.7)	9 (6.1)	1 (5.9)	25 (7.4)
Former smoker	52 (6.6)	3 (1.0)	4 (2.7)	0 (0.0)	45 (13.4)
Never smoked	700 (88.4)	283 (97.3)	135 (91.2)	16 (94.1)	266 (79.2)
Alcohol drinking experience, available *n* (%) ^§^	612 (46.8)	195 (41.4)	87 (23.1)	1 (2.9)	329 (77.4)
Drinker, *n* (%)	383 (62.6)	16 (8.2)	48 (55.2)	0 (0.0)	319 (97.0)
Non-drinker	229 (37.4)	179 (91.8)	39 (44.8)	1 (100.0)	10 (3.0)
Body mass index, available *n* (%) ^§^	452 (34.6)	188 (39.9)	108 (28.7)	9 (25.7)	147 (34.6)
<18.5 kg/m^2^, *n* (%)	114 (25.2)	61 (32.4)	32 (29.6)	3 (33.3)	18 (12.2)
18.5–24.9	266 (58.8)	96 (51.1)	63 (58.3)	4 (44.4)	103 (70.1)
25.0–29.9	68 (15.0)	30 (16.0)	11 (10.2)	2 (22.2)	25 (17.0)
≥30	4 (0.9)	1 (0.5)	2 (1.9)	0 (0.0)	1 (0.7)
Family history of IBD, yes *n* (%) ^¶^	29 (2.2)	11 (2.3)	8 (2.1)	2 (5.7)	8 (1.9)
Any comorbidity at enrollment, *n* (%) ^¶^	494 (37.8)	212 (45.0)	98 (26.1)	10 (28.6)	174 (40.9)
Cancer	51 (10.3)	5 (2.4)	5 (5.1)	0 (0)	41 (23.6)
Non-cancer	443 (89.7)	207 (97.6)	93 (94.9)	10 (100.0)	133 (76.4)
PCDAI, available *n* (%) *	—	360 (76.4)	—	—	—
≤10, *n* (%)	—	118 (32.8)	—	—	—
11–30	—	58 (16.1)	—	—	—
>30	—	184 (51.1)	—	—	—
CDAI, available *n* (%) *	—	51 (10.8)	—	—	—
<150, *n* (%)	—	17 (33.3)	—	—	—
150–219	—	3 (5.9)	—	—	—
220–450	—	30 (58.8)	—	—	—
>450	—	1 (2.0)	—	—	—

CD, Crohn’s disease; UC, ulcerative colitis; Other GE/colitis, other non-infective gastroenteritis and colitis; CRC, colorectal cancer; IBD, inflammatory bowel disease; PCDAI, pediatric Crohn’s disease activity index; CDAI, Crohn’s disease activity index. ^†^ Mean ± standard deviation of age at diagnosis, calculated from available records. ^§^ Number (%) of participants in each group with recorded data. Category percentages use the number of participants with available data as the denominator. Missing data were not imputed. Body mass index was calculated from height and weight extracted in bulk from the hospital clinical data warehouse. The same BMI categories are presented descriptively for all ages and should not be interpreted as pediatric weight-status classifications. Alcohol data were available for only one participant with Other GE/colitis. ^¶^ A positive family history of IBD was documented for 29 participants. Percentages use the total number of participants in each group as the denominator. Missing family history information was not classified as a negative history. Percentages for any comorbidity use all participants in each group as the denominator; percentages for cancer and non-cancer comorbidities use participants with any recorded comorbidity in that group as the denominator. * Reported for participants with CD only. The PCDAI was applied to participants younger than 18 years and the CDAI to those aged 18 years or older. Availability is shown as a percentage of the 471 participants with CD; category percentages use the number of participants with available data.

**Table 6:** Availability of medical imaging and digital pathology data among 1307 participants, 2021–2025.

Type	2021	2022	2023	2024	2025	Total
**Endoscopy**	103	143	230	76	37	589
**X-ray**	─	─	─	28	15	43
**CT**	27	29	116	60	48	280
**MRI**	47	68	142	35	16	308
**U** **ltrasonography**	9	2	─	6	5	22
**Digital pathology**	─	─	225	126	112	463

Values indicate the number of participants with available data for each modality among the 1307 enrolled participants. Because individual participants may have data across multiple modalities, counts should not be summed to calculate the total number of participants.

**Table 7:** Annual bioresource distribution by approved applications and vials, 2021–2025.

Type	2021	2022	2023	2024	2025	Total
**IBD and CRC**	4 cases ^†^ (752 vials)	6 cases (86 vials)	10 cases (658 vials)	7 cases (106 vials)	7 cases (798 vials)	34 cases (2400 vials)
**Bottom-up collection**	8 cases (1287 vials)	17 cases (954 vials)	16 cases (2788 vials)	12 cases (2032 vials)	22 cases (3313 vials)	75 cases (10,374 vials)

^†^ A distribution case is defined as a single approved application for bioresource utilization. Vial counts represent the number of distributed vials per calendar year, with the total reflecting the sum of annual counts.

**Table 8:** Research topics utilizing bioresources and types of bioresources distributed in the biobank.

Categories	Research Subject	Cases *	Bioresource
Disease diagnosis and prediction	Biomarker development	37	BLD, BUF, PLA, SER, STL, TSU, CEI, MID
	Nucleic acid polymorphism analysis	4	SER, TSU
	Disease diagnostic technology	23	BLD, BUF, PLA, SER, TSU, URN, CEI, MID
Therapeutic development	Targeted therapeutics development	10	PLA, SER, STL, TSU
	Microbial analysis	4	SER, STL
Disease mechanisms and pathogenesis	Disease model development	15	PLA, SER, STL, TSU, URN
	Disease-related gene and protein function	24	BLD, PLA, SER, TSU, CEI
	Epigenomic research	1	TSU
Public health and epidemiology	Infectious disease surveillance and response	2	PLA, SER
Advanced technology	Machine learning, deep learning, etc.	7	BLD, PLA, SER, CEI
Others	Human bioresource quality control	5	BLD, TSU
	Forensics	1	BLD
	Diet and nutrition	1	SER

BLD, whole blood; BUF, buffy coat; PLA, plasma; SER, serum; STL, stool; TSU, tissue; URN, urine; CEI, clinical and epidemiological information; MID, medical imaging data. * Counts represent the number of approved distribution applications (*n* = 109 in total: 34 for IBD- and CRC-related bioresources and 75 for the bottom-up collection). Because individual applications could cover multiple research topics, the topic-specific counts sum to 134 across 109 approved applications.

## Data Availability

Access to the data and biospecimens is available upon reasonable request, subject to formal application and approval by the KNUH Biobank team currently conducting the fifth phase of the project under a distinct team of investigators.

## Abbreviations

The following abbreviations are used in this manuscript:

AI	Artificial intelligence
API	Application programming interface
BMI	Body mass index
BRIDGE	Biobank Research Information and Digital Image Exchange
CD	Crohn’s disease
CDAI	Crohn’s disease activity index
CDM	Common data model
CRC	Colorectal cancer
CT	Computed tomography
ETL	Extract, transform, load
H&E	Hematoxylin and eosin
HCI	Hyperconverged infrastructure
IBD	Inflammatory bowel disease
IRB	Institutional review board
ISBER	International Society for Biological and Environmental Repositories
KASID	Korean Association for the Study of Intestinal Diseases
KBN	Korea Biobank Network
KCD	Korean Standard Classification of Diseases
KNIH	Korea National Institute of Health
KNUCH	Kyungpook National University Chilgok Hospital
KNUH	Kyungpook National University Hospital
MRI	Magnetic resonance imaging
ODS	Operational data store
Other GE/colitis	Other non-infective gastroenteritis and colitis
PACS	Picture archiving and communication system
PCDAI	Pediatric Crohn’s disease activity index
PCR	Polymerase chain reaction
QC	Quality control
RIN	RNA integrity number
SOP	Standard operating procedure
SPREC	Standard PREanalytical Code
UC	Ulcerative colitis
